# Patent Foramen Ovale (PFO), Personality Traits, and Iterative Decompression Sickness. Retrospective Analysis of 209 Cases

**DOI:** 10.3389/fpsyg.2017.01328

**Published:** 2017-08-02

**Authors:** Pierre Lafère, Costantino Balestra, Dirk Caers, Peter Germonpré

**Affiliations:** ^1^Laboratoire ORPHY—EA4324, Université de Bretagne Occidentale Brest, France; ^2^Research and Education, Divers Alert Network Europe Rosetto, Italy; ^3^Anatomical Research and Clinical Studies, Vrije Universiteit Brussel Brussels, Belgium; ^4^Motor Sciences, Université libre de Bruxelles Brussels, Belgium; ^5^Anatomical Research Training and Education, Vrije Universiteit Brussel Brussels, Belgium; ^6^Environmental, Occupational, Ageing (Integrative) Physiology Laboratory, Haute Ecole Bruxelles-Brabant Brussels, Belgium; ^7^Centre for Hyperbaric Oxygen Therapy, Military Hospital Queen Astrid Brussels, Belgium

**Keywords:** peer review, health care, diving, risk-taking, prevention, accident

## Abstract

**Introduction:** There is a need to evaluate the influence of risk factors such as patency of foramen ovale (PFO) or “daredevil” psychological profile on contra-indication policy after a decompression sickness (DCS).

**Methods:** By crossing information obtained from Belgian Hyperbaric Centers, DAN Emergency Hotline, the press, and Internet diving forums, it was possible to be accountable for the majority if not all DCS, which have occurred in Belgium from January 1993 to June 2013. From the available 594 records we excluded all cases with tentative diagnosis, medullary DCS or unreliability of reported dive profile, leaving 209 divers records with cerebral DCS for analysis. Demographics, dive parameters, and PFO grading were recorded. Twenty-three injured divers were tested using the Zuckerman's Sensation Seeking Scale V and compared to a matched group not involved in risky activities.

**Results:** 41.2% of all injured came for iterative DCS. The average depth significantly increases with previous occurrences of DCS (1st DCS: 31.8 ± 7.9 mfw; 2nd DCS: 35.5 ± 9.8 mfw; 3rd DCS: 43.4 ± 6.1 mfw). There is also an increase of PFO prevalence among multiple injured divers (1st DCS: 66.4% 2nd & 3rd DCS: 100%) with a significant increase in PFO grade. Multiple-times injured significantly scored higher than control group on thrill and adventure seeking (TAS), experience seeking, boredom susceptibility and total score.

**Conclusion:** There is an inability of injured diver to adopt conservative dive profile after a DCS. Further work is needed to ascertain whether selected personality characteristics or PFO should be taken into account in the clearance decision to resume diving.

## Introduction

Upon their ascent and in the hours after the dive, SCUBA divers expose themselves to possible nitrogen decompression problems. These problems (DCS: Decompression Sickness) are caused by gas bubbles formation in the blood vessels and/or supersaturated body tissues (Germonpre et al., 2015). Although, the precise mechanisms are not known, many provocating factors have been advocated in bubble formation or consequences (Carturan et al., 1999; Blatteau et al., 2008; Germonpre et al., 2009).

One of them, the Patency of Foramen Ovale (PFO), a condition that is present in about one third of the human population is a heritage of the fetal cardiac circulation (Hagen et al., 1984). It has been well associated to certain forms of DCS. Indeed, PFO is a pathway through which vascular gas emboli (VGE) can arterialize, given sufficiently favorable circumstances (such as large amount of VGE, PFO grading, straining maneuvers, delayed desaturation, etc.). Therefore, it seems to be a direct relationship between “cerebral” forms of DCS and PFO (Balestra et al., 1998, 2004; Germonpre et al., 1998; Ries et al., 1999; Cantais et al., 2003; Mitchell and Doolette, 2009; Wilmshurst et al., 2015).

Nonetheless, routine screening for PFO at the time of dive medical fitness assessment (either initial or periodic) is not indicated. However, consideration should be given to investigating for PFO if the diver has suffered from DCS, especially if the dive profile was not very ≪ provocative ≫ and if the DCS was characterized by cerebral, spinal, vestibulocochlear, or cutaneous manifestations (UHMS, 2011; Smart et al., 2015).

After the diagnosis of a PFO, considered likely to be associated with increased DCS risk (Odds Ratio Between 2.5 and 5.6; Bove, 1998; Germonpre et al., 1998; Torti et al., 2004), the diver may consider several options in consultation with a diving physician such as quitting diving, diving more conservatively (Examples include: reducing dive times to well inside accepted no-decompression limits; restricting dive depths to <30 m; performing only one dive per day; use of nitrox with air dive planning tools; intentional lengthening of a safety stop or decompression time at shallow stops; avoidance of heavy exercise and unnecessary lifting or straining for at least 3 h after diving, etc.) or PFO closure (Billinger et al., 2011).

This study aimed to examine the willingness of experienced recreational scuba divers in Belgium to comply with more conservative diving procedure after an initial DCS and a positive PFO diagnosis. These data will assist in evaluating the effectiveness of the medical counseling after a DCS, and the need for possibly stricter contra-indication related to “daredevil” psychological profile.

## Methods

Belgium counts about 10 hyperbaric centers, however only three of them have the expertise to treat injured divers: Antwerp (UZA), Brussels (Military hospital), and Charleroi (Civil hospital). This regional dispersion does not facilitate the data gathering on diving accidents. However, by crossing information obtained from the Hyperbaric Centers of Brussels and Charleroi, the DAN Emergency Hotline, the press and Internet diving forum's, it was possible to be accountable for the majority if not all DCS, which have occurred in Belgium from January 1993 to June 2013.

Although, full ethical review and approval was not required for this study in accordance with the national and institutional requirements, all 594 Belgian divers who suffered from DCS were reviewed in accordance with the Declaration of Helsinki. Each diver gave verbal consent for use of their case in studies where only group data are reported. In this study, when a case was identified for inclusion, the clinical information was loaded into a database that was stripped of individual identifiers.

For all the divers, we recorded several data such as age, gender, diving certification, number of dives performed, years of experience, previous history of dive accident, type of accident, circumstances of the accident, and presence of a PFO with grading (grade 0, no contrast passage at rest or after Valsalva strain; grade 1, no or slight (<20 bubbles) contrast passage at rest or after Valsalva strain; and grade 2, important (≥20 bubbles) contrast passage at rest or after Valsalva strain; Germonpre et al., 1998).

The majority of these cases are consulting with neurological symptoms. Cases with tentative diagnosis (minor, vague, and subjective symptoms not responding to proper treatment), medullary DCS (no significant correlation between the prevalence of PFO and the occurrence of spinal DCS; Germonpre et al., 2015; Balestra and Germonpre, 2016) or unreliability of reported dive profile were therefore excluded.

This left 286 divers records with cerebral DCS for analysis. From these, 239 medical files were available but only 209 contained all the necessary information for analysis.

From 2010 to 2013, 23 injured divers were tested with the Zuckerman's Sensation Seeking Scale V (Zuckerman, 1983). It has indeed been used in several studies to identify the sensation seeking and risk taking traits in risk sports (Dahlback, 1990; Cronin, 1991; Freixanet, 1991; Harding and Gee, 2008). Form V of the scale which is most used operates with a total scale and four subscales: thrill and adventure seeking (TAS), experience seeking (ES), disinhibition (Dis), and boredom susceptibility (BS). We used a French translation of Zuckerman's Sensation Seeking Scale. The translation was done by Carton et al in 1990 and tested for reliability and factor structure (Carton et al., 1990). They were then compared to a matched group of individual not involved in risky activities.

### Statistical analysis

Clinical recovery after 6 month (complete, mild or severe residual symptoms) and PFO grading were considered as a dependent variable and were analyzed using nonparametric testing of the difference in ranks. Characteristics related to the dive and clinical parameters were analyzed as independent variables and were analyzed with unpaired *t*-Test or repeated-measures ANOVA with Bonferroni *post-hoc*. All data passed the Kolmogorov-Smirnov test, allowing us to assume a Gaussian distribution.

GraphPad Prism version 5.00 for Windows (GraphPad Software, San Diego, California, USA) was used as standard computer statistical package., A threshold of *P* < 0.05 was considered statistically significant. Data are presented as mean ± standard deviation (*SD*) unless precised otherwise.

## Results

In our sample, injured divers are mostly men (80.4%) being 40.5 ± 11.2 years old (15–68 years, median 42.3 years) with a dive experience of 9.6 ± 9.3 years (0–37 years, median 6 years) and 646 ± 101 dives (5–2,959 dives, median 300 dives).

Incident breakdown by diver qualification shows that no certification level, from novice to instructors, is immune to problems. When compared to an historical cohort (1995–2005; Lafere et al., 2009), instructors are overrepresented (26.9% of the injured divers vs. 8.6% of the diving population, One Way ANOVA, *p* < 0.01).

Ninety-six percent of the recorded dives were performed in flooded quarries and gravel pits as well as in dam's reservoirs [i.e., Fresh water depth (mfw)]. Although, there is no cave diving, visibility varies greatly depending on the quarry (between 1 and 10 m), the number of divers, and the nature of bottom, marble quarries being the clearest. Under the thermocline, which depth varies between 1 and 10 m according to the season, the temperature oscillates between 1 and 8°C all year. Above, the temperature oscillates between 1°C in winter and 18°C in the summer.

Dive profile and decompression were managed either according to a personnal dive computer (168/209—80.38%), US Navy dive table with timer and depth gauges (30/209—14.35%) or a customized dive table generated by a decompression software (11/209—5.26%). The two main used decompression models were the Uwatec™ Bühlmann ZH-L8 ADT and the Suunto™ RGBM. However, statistical analysis fail to demonstrated any difference in DCS severity or outcome between these two decompression models. Dives were characterized by a maximal depth of 33.8 ± 9 mfw (14–60 mfw, median 34 mfw) and a total dive time of 39.5 ± 13 min (13–89 min, median 39 min). From the 87 available dive-profile printouts, we observed a dive profile error in 40% of cases (normal saturation/inadequate offgassing, mainly due to low/out-of-air situation due to poor planning), a “logical” cause of decompression incident in 20% of cases (increased saturation/“normal” offgassing or increased or “normal” saturation/insufficient offgassing as in cases of strenuous effort or cold water diving) and finally in 40% of the cases the accident is declared undeserved. These dive parameters or profile errors seem to depend much more on the level of acquired skills, than on the teaching system as no statistical difference in DCS severity or outcome was observed between PADI vs. CMAS (One Way ANOVA, *p* = 0.488).

Without knowing the number of dives carried out, it is difficult to calculate the incidence of the accidents. However, from a previous study (Lafere et al., 2009), this number was estimated at 1,042,618 dives per year. This gives us an estimated incidence for DCS in Belgium of 0.73/10,000 dives.

From the 209 injured diver records analyzed, 125 (59.8%) were treated for a first episode of cerebral DCS (1st DCS), 70 (33.5%) for a second episode (2nd DCS) and 14 (6.7%) for a third one (3rd DCS). With regard to biometric data (age, body mass index), smoking habits, no significant differences were found between DCS subgroups. However, there was a significant difference in the number of dive done each year between the “first-time” and the “multiple-times” injured divers, which seem to be the more active. Applied treatment did not differ between groups but for the number of additional hyperbaric oxygen session (HBOT). Outcomes are resumed in Table 1.

**Table 1:** Long-term outcomes of 209 divers with cerebral decompression sickness.

		**1st DCS (*n* = 125)**	**2nd DCS (*n* = 70)**	**3rd DCS (*n* = 14)**	***p***
**Dives/year**		40 ± 2.7 [5–168, median 34]	70 ± 8.1 [25–200, median 60]	74 ± 8.9 [20–125, median 77]	<0.0001
**Treatment received**		1 ± 0.07 USN TT6 [0–2, median 1]	1 ± 0.14 USN TT6 [0–2, median 1]	1 ± 0.17 USN TT6 [0–2, median 1]	0.75
		7 ± 0.8 HBOT [0–20, median 6]	11 ± 1.3 HBOT [4–18, median 10]	8 ± 1 HBOT [2–12, median 8]	0.04
**Outcome**	**Complete resolution**	73.6%	27.1%	0%	
	**Mild residual symptoms**	19.2%	57.1%	85.7%	0.08
	**Severe residual symptoms**	7.2%	15.7%	14.3%	
**Resume diving**		81.6%	84.3%	0%	NA
**Delay to resume diving**		4.2 ± 0.7 months [0.5–15, median 5]	3.5 ± 0.6 months [1–8, median 5.5]	NA	0.97

*Mild residual symptoms are mild paresthesia, weakness, residual pain or some impairment of daily activities. Severe residual symptoms are difficulty walking, paralysis, uncompensated vertigo, or speech disorders. USN TT6, US Navy Treatment Table 6, i.e., 2.8 ATA, 100% oxygen for 285 min with air break. HBOT, Hyperbaric oxygen session 2.5 ATA, 100% of oxygen for 70 min without air break. Data are presented as mean ± standard error on mean (SEM)*.

**Figure 1:**
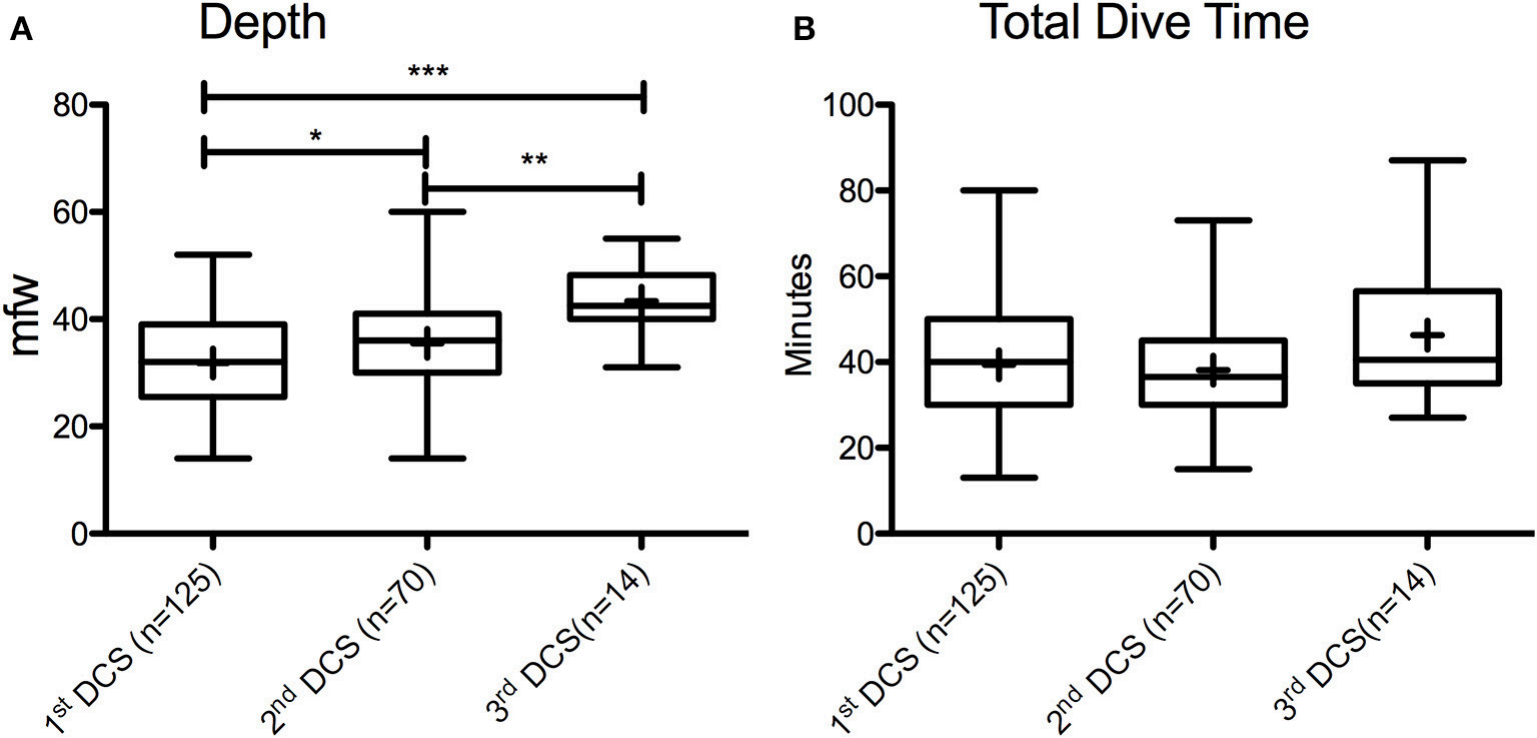
Box and whisker plots of variation in dive parameters (**A**. Depth; **B**. Total dive time) according to decompression sickness occurrence (DCS), indicating median, 25–75 percentiles and minimum and maximum observations; “+” marked in the boxes indicate the means; ^*^p < 0.05; ^**^p < 0.01; ^***^p < 0.001.

It is important to note that 41.2% of all injured came for iterative DCS. Further analysis Figure 1 shows that the average depth of the causal dives significantly increases with the previous occurrences of DCS (1st DCS: 31.8 ± 7.9 mfw; 2nd DCS: 35.5 ± 9.8 mfw; 3rd DCS: 43.4 ± 6.1 mfw; *p* < 0.0001). Total dive time is not different (1st DCS: 39 ± 13 min; 2nd DCS: 38 ± 11 min; 3rd DCS: 46 ± 16 min; *p* = 0.458).

**Figure 2:**
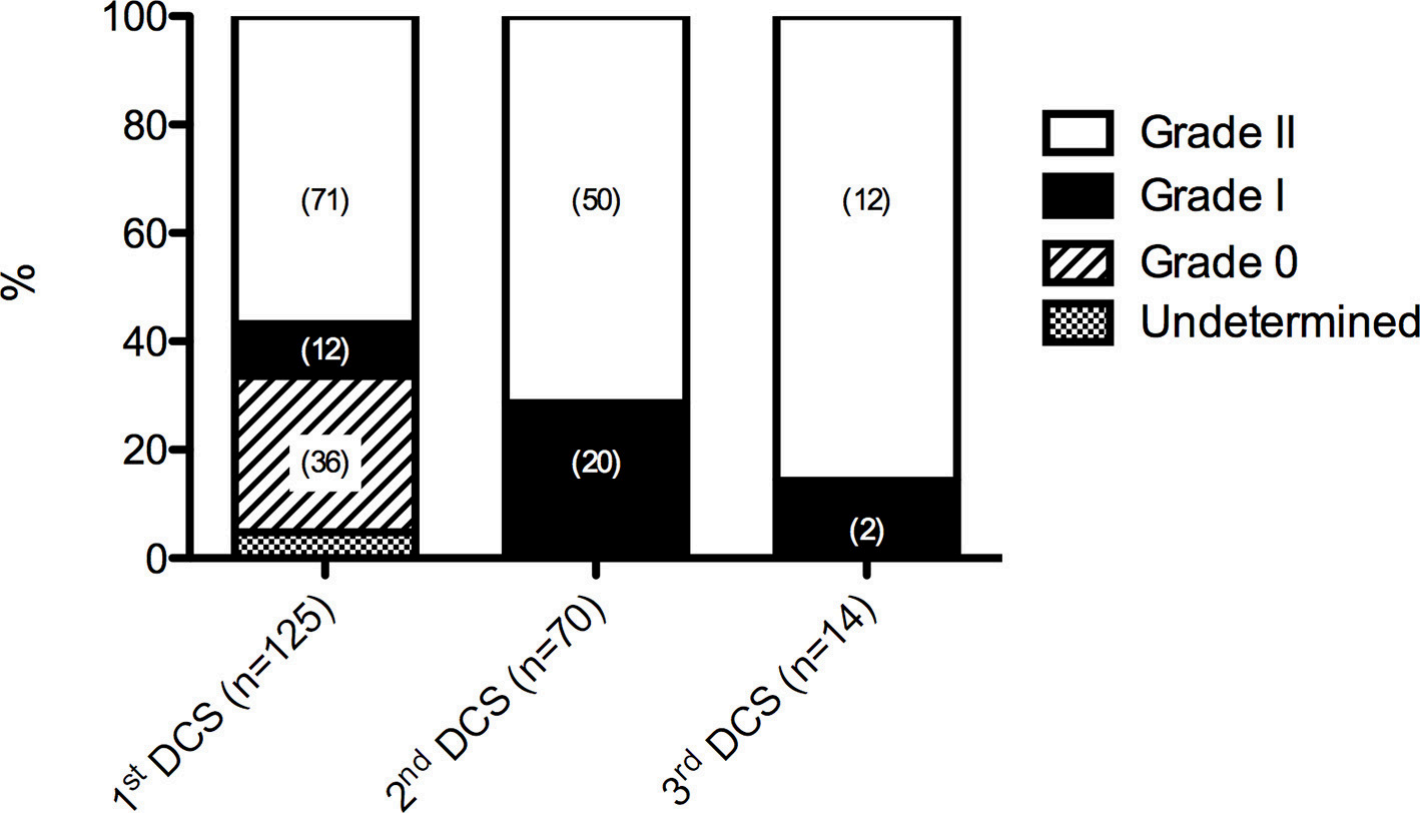
Prevalence of PFO (patent foramen ovale). Grade 0, no PFO—no contrast passage at rest or after Valsalva strain; Grade 1, no or slight (<20 bubbles) contrast passage at rest or after Valsalva strain; and Grade 2, important (≥20 bubbles) contrast passage at rest or after Valsalva strain. DCS, decompression sickness. Numbers in parentheses are total number of cases.

All divers underwent echocardiography, either transthoracic (TTE) or transesophageal (TEE), with the use of agitated saline for contrast in order to assess the presence or absence of patency of the foramen ovale. The prevalence of PFO in these groups (Figure 2) increased with the occurrence of iterative DCS (1st DCS: 66.4% 2nd & 3rd DCS: 100%). Also, at the second and third DCS, all injured divers had increased their permeability, from zero to grade 1 or 2 PFO or from 1 to grade 2 PFOs. The difference of TEE/TTE score on an ordinal scale was statistically significant (Wilcoxon signed rank test, *p* = 0.023).

On the Sensation Seeking Scale V, multiple injured scored higher than all other groups on TAS, ES, BS, and Total score (Table 2). The differences were significant in relation to control group on all scales except for Dis. Between the two diver groups only TAS difference was significant.

**Table 2:** Sensation seeking scale V mean scores of divers with multiple DCS (*n* = 7), compared with first timer DCS (*n* = 16) and control group (*n* = 23).

	**1st DCS (*n* = 16)**	**Multiple DCS (*n* = 7)**	**Control group (*n* = 23)**	***p***	**1st DCS vs. control**	**Multiple DCS vs. control**	**1st DCS vs. Multiple DCS**
TAS	7.44 ± 2.03	8.71 ± 2.03	4.35 ± 1.59	<0.001	<0.001	<0.001	<0.01
ES	6.06 ± 1.77	7.71 ± 1.78	5.31 ± 2.10	<0.01	<0.05	<0.01	ns
Dis	5.22 ± 2.41	4.85 ± 1.95	4.7 ± 1.87	0.816	ns	ns	ns
BS	4.44 ± 1.79	5.71 ± 1.98	4.26 ± 1.96	<0.05	<0.05	<0.05	ns
Total	21.7 ± 6.8	27 ± 4.2	18.4 ± 3.7	<0.01	<0.05	<0.01	<0.01

*Total score correspond to the sum of subscale: thrill and adventure seeking (TAS), experience seeking (ES), disinhibition (Dis), and boredom susceptibility (BS)*.

## Discussion

One of the most remarkable observation was undoubtedly that despite all divers receiving medical counseling about diving safety and DCS prevention when resuming diving (consisting of a 1 h consultation with schematic drawing of PFO and bubble possible pathways and risks), more than one third of the diver were admitted for iterative DCS. This might be explained by two hypotheses.

First, there is the legitimate question of either the effectiveness of preventive measures, or the implementation of these measures when resuming diving after an accident. On one hand, studies that have evaluated procedures to reduce nitrogen load after a first episode of DCS appeared to reduce the probability for subsequent DCS (Klingmann et al., 2012; Honek et al., 2014a,b). On the other hand, it has to be noted that subgroup analysis shows that multiple time injured divers dived significantly more than the average diver with 70 ± 38 dives/year and 74 ± 33 dives/year for 2nd DCS and 3rd DCS group, respectively. Moreover, the average depth of the causal dives significantly increases with subsequent occurrences of DCS (1st DCS: from 31.8 ± 7.9 mfw for the first DCS to 43.4 ± 6.1 mfw for the third DCS). This suggests that having had a decompression accident does not seem to constitute a sufficient argument to modify a diver's underwater behavior.

Indeed, whereas all sportsmen seek physical sensations, they not necessarily do so by voluntary adopting behaviors known to be dangerous (Lafollie and Le Scanff, 2007). However, injured divers score very high not only on TAS but also on ES, which means that they are eager to seek new unusual experiences in all areas of life. Divers also score unusually high on boredom susceptibility. On the disinhibition scale there are no differences with control. High disinhibition scorers enjoy partying but are not willing to take the risk of making a fool of oneself or becoming a social misfit. The high TAS and ES scorers may be more used to take risks. For high TAS scorers there is a constant risk of severe injury or death. For high ES scorers there may be the risk of becoming a drug addict and the social consequences that go with it (Breivik, 1997). Although, the number of multiple injured divers is small, they scored significantly higher on TAS than any other groups, giving support for the notion of physical risk taking. Indeed, many diving accidents are at least in part attributable to failure to follow correct procedures. As has been stated for over 50 years in the British Subaqua Club incident report: “most of the incidents reported within this document could have been avoided had those involved followed a few basic principles of safe diving practice” (BSAC, 2016). However as Harding & Gee mention in their study, the preferred way to examine the role of personality as a predisposing factor in DCS would be to measure variables before as well as after the incident. Without such data the possibility of the experience having an effect on supposedly stable personality characteristics cannot be ruled out (Harding and Gee, 2008). Nonetheless, one observation may confirm the importance of behavioral issues. Indeed, the monthly breakdown of accidents shows that Belgian diver dives all year round independently of the season. As in other databases, BSAC for example, 50% of the reported incidents have occurred in the summer period (June–September), however with an unusual January peak in the winter period. This anomaly can possibly be explained either by a more significant number of divers continuing their activity during the winter months, or more probably by the more rigorous winter we had in 1997, 1998, 2009, 2010, 2012 compared to other years. Indeed, when these years are excluded, distribution follows a Gaussian pattern. When planning a dive in cold water or in conditions that might be strenuous, dive tables requires the divers to assume a depth that is 3 m deeper than the actual depth. Nonetheless none of the injured divers during this particular period have adapted their decompression schedules. Another argument seems to confirm the importance of behavior. Normally one star divers are limited in depth. Indeed, in Belgium, they cannot dive deeper than 15 m (20 m when accompanied by an instructor). Yet the average depth of their accidents is 24.1 ± 9.1 mfw (17–43, median 24). In the same way, the maximum depth allowed in quarry, gravel pit and lake's reservoirs is 40 m pushed for seasoned divers (four stars divers and instructors) to 60 m in case of air diving. Yet the average depth of instructor accidents is 41.4 ± 9.7 mfw (27–69, median 40.5). These faulty dive profiles may reveal some hidden psychological motive or a potential self-destructive attitude questioning diver's capacity to understand and to cope with specific risk.

The second hypothesis relies with the patency of foramen ovale (PFO). Our results show an increase of PFO prevalence among multiple injured divers with furthermore, also an increase in PFO grade. Since this is a retrospective study, a selection bias cannot be fully excluded, which would mean our results are just an incidental finding. However, there are arguments why this would not be the case. Indeed, a statistical correlation has been shown between ischemic cerebral incidents in diving (cerebral DCS) and large PFOs (grade 2). The same is true for PFO and “unexplained” stroke (Van Camp et al., 1993; McGaw and Harper, 2001). No such correlation has been demonstrated for small PFOs (grade 1). Moreover, a prospective follow-up study has documented the increase in PFO size in humans (Germonpre et al., 2005). This is an important finding, as the authors stated it, because it may imply that increased susceptibility to neurologic DCS could develop over time. According to our results this seems to be the case.

Finally, although not statistically significant, it has to be noted that the risk of residual symptoms, mild or severe, seems to increase with the number of DCS. This might be explained by several mechanisms. The more frequently, and the more deep you dive, the more serious DCS one risks; alternatively, there may be a depletion of the physiological “reserve neurological capacity” with each new accident, the potential for healing being reduced every time. This can explained why injured divers focus on the simplistic idea that they need “to get fixed” and why technical diving organizations even have recommended preventive PFO closure in order to undertake high-risk dive training. Anecdotally, it should be noted that two divers of our series had benefited from a PFO closure within the 10 years preceding their second accident. During the ultrasound control, they both had a grade 2 PFO despite the device being in place. Studies with long-term follow-up of PFO closure among divers therefore appear mandatory. In the meantime, safe diving is something to be learned, not something that can be implanted (Germonpre, 2015).

There are some inherent limitations to this study, mainly concerning the representativeness of the divers in our database. First, we cannot be sure that we do not have a full record of all types of incident. If a hyperbaric center is not involved and if those involved do not declare their accident, then it will go unrecorded. It is impossible to assess just how many incidents are unrecorded.

Although, not the only sports divers' federation in Belgium, the Belgian chapter of CMAS (FEBRAS) is by far the largest group, and 83.3% of the divers from our database are affiliated with them. There are many similarities between the two populations. First, the average age is similar (40.5 ± 11.2 vs. 40.6 ± 11 years). Secondly, from the data obtained from our patients, the accident victims performed a total of 65,134 dives over a cumulative period of 1,063 years, yielding to an average number of 51 ± 45 dives/year (5–200, median 45). Based on retrospective data obtained from CMAS affiliated dive clubs, this number is consistent with the Belgian Underwater Federation estimation of 45–50 dives/year.

The certifications breakdown between the two populations is also very similar. There are some differences, as our database does not contain any divers with no certification and has a significant over representation of instructors. This might be explained by the fact that instructors carried out the greatest number of dives by far [64 ± 42 dives/year, (5–200, median 95)] as well as the deepest dives [39.8 ± 11.3 mfw, (24–61, median 37)]. This seems also logical, as seasoned divers, who naturally achieve higher ranking in their respective organizations through the years, constitute a large part of the examined cohort.

Finally, women constitute 19.6% of the cohort, from novice to instructor. Unfortunately, we do not know the proportion of woman affiliated to the FEBRAS. However, this figure is coherent with the literature, several commercial studies having shown, women fluctuate 1 year on the other around 25% of all active divers (Shapiro, 2003; Altman et al., 2005).

This is why this report should be treated as a sample and not as a definitive and complete record. However it gives a fair picture of the injured diver, dive parameters, risk factors and outcome in Belgium, which seems representative of the whole diving population.

Diver education toward save diving through adoption of conservative dive profiles to significantly reduce the risk of recurrent DCS is of paramount importance (Klingmann et al., 2012). However, from the results of the present study, it seems obvious that further work is needed to ascertain whether selected personality characteristics should be taken into account in the clearance decision to resume diving after a DCS because PFO remains a reason for caution where definitive recommendations still cannot be made (Germonpre, 2015).

Although, PFO is considered a risk factor for cerebral DCS in SCUBA divers, the primary cause of DCS, however, is the nitrogen bubble, not the PFO (Germonpre, 2005; Germonpre et al., 2005). Therefore the degrees of DCS risk reduction dependent on how the diver manages his/her dive and decompression to reduce the incidence of VGE (Germonpre, 2015) which depends on the behavioral capacity to comply with more conservative dive profile. From the present study, it is clear that diving safety is something to be learned.

## Author contributions

DC and PL provided substantial contributions to the conception and design of the work; and the acquisition, analysis, and interpretation of data for the work. PL has drafted the work and revised it critically for important intellectual content. PG and CB revised it critically for important intellectual content. Final approval of the version to be published was the responsibility of PL, PG, and CB.

### Conflict of interest statement

The authors declare that the research was conducted in the absence of any commercial or financial relationships that could be construed as a potential conflict of interest. The handling Editor declared a shared affiliation, though no other collaboration, with one of the authors CB and states that the process nevertheless met the standards of a fair and objective review.
